# Evaluation of intraoperative touch imprint cytology on axillary sentinel lymph nodes in invasive breast carcinomas, a retrospective study of 1227 patients comparing sensitivity in the different tumor subtypes

**DOI:** 10.1371/journal.pone.0195560

**Published:** 2018-04-12

**Authors:** Hafsteinn Ingi Pétursson, Anikó Kovács, Jan Mattsson, Roger Olofsson Bagge

**Affiliations:** 1 Department of Surgery, Institute of Clinical Sciences, Sahlgrenska Academy at University of Gothenburg, Sahlgrenska University Hospital, Gothenburg, Sweden; 2 Department of Clinical Pathology and Genetics, Sahlgrenska University Hospital, Gothenburg, Sweden; University of North Carolina at Chapel Hill School of Medicine, UNITED STATES

## Abstract

**Background:**

Intraoperative evaluation of the axillary sentinel lymph node (SLN) in patients with breast carcinoma reduces the need of re-operations in cases where an axillary completion lymph node dissection (CLND) is indicated. Different methods have been used to determine the SLN status intraoperatively, e.g. frozen section histology (FS) and touch imprint cytology (TIC). The sensitivity of intraoperative TIC examination on SLN is not consistent between different studies and varies according to different tumor histologic subtypes, tumor size and the age of the patient. The aim of this study was to describe the specificity and sensitivity of TIC and to compare TIC sensitivity in the different histological subtypes of breast carcinoma.

**Methods:**

A retrospective review was performed of 1227 consecutive clinically node negative breast cancer patients treated with sentinel lymph node biopsy (SLNB) with intraoperative TIC between the years 2003 and 2008. The SLN was bisected and stained using the May-Grünwald-Giemsa method and immunocytochemically with the antibody MNF-116.

**Results:**

The overall sensitivity of the TIC test was 68.6% and the specificity was 99.8%. There was no statistically significant difference between the detection of SLN metastases from ductal carcinoma versus lobular carcinoma. The sensitivity improved over the period of the study.

**Conclusion:**

TIC is highly specific with an acceptable overall sensitivity. The sensitivity increased under the period of the study and it was higher in cases with larger size of the primary tumor. There was no difference in TIC sensitivity between the different histological subtypes.

## Introduction

The sentinel lymph node biopsy (SLNB) technique was developed in the 1990s by Donald Morton et al [1]. This technique is used to detect the spread of invasive tumor cells from the primary tumor to regional lymph nodes. Intraoperative evaluation of the sentinel lymph node (SLN) status is desirable because patients with positive status can undergo a completion lymph-node dissection (CLND) in the same procedure, reducing the need for another operation at a later date. Furthermore, patients who have a negative SLN status would not need any further surgery, thus avoiding potential complications from an axillary lymph node dissection.

Diverse methods have been used to determine SLN status intraoperatively, e.g. frozen section histology (FS) [2], touch imprint cytology (TIC) [3], immunohistochemistry [4] and infrared spectroscopy [5]. TIC is considered a more tissue conserving and less expensive method compared to FS with comparable accuracy [6]. The sensitivity of the TIC test on SLN in breast cancer is not consistent between studies, and seems to vary according to different tumor types, tumor size and the age of the patient [4–10]. Especially metastasis of invasive lobular carcinoma in the SLN is reported to be more difficult to identify on pathological examination compared to ductal carcinoma. Lobular carcinoma more often have a low-grade cytomorphology and a tendency to infiltrate lymph nodes in a single cell pattern, thus resembling lymphocytes [7,8].

The aim of this study is to evaluate the sensitivity and specificity of TIC in a consecutive series of breast cancer patients undergoing SLNB, with a special focus on how the sensitivity varies between tumor size and histological subtype.

## Methods

### Patients

A retrospective review was performed of a prospectively maintained database of all consecutive SLN cases with intraoperative TIC for breast cancer and clinically negative axillary lymph nodes between October 2003 and December 2008. All cases with invasive breast carcinoma were included (n = 1571), however patients with a diagnosis of bilateral breast carcinoma (n = 25), a previous diagnose of invasive breast carcinoma (n = 112), a benign pathology of the primary tumor on final histopathologic examination (n = 202) as well as males with breast carcinoma (n = 5) were excluded. A total of 1227 patients were finally eligible for analysis. A chart review of each SLN case was performed for the following data: age, year of diagnosis, type of breast surgery and type of axillary surgical procedure. Primary tumor data included: size, histologic subtype, histologic grade and hormone receptor status. Stage was defined according to the guidelines of the American Joint Committee on Cancer (AJCC) 7th edition. The research was approved by the ethics committee of the Sahlgrenska University Hospital. The need for consent was waived by the ethics committee.

### Surgical protocol

A double modality technique was used to identify the SLN. First the breast skin was injected with technetium 99m (0.5–1.0 mCi) labelled human albumin colloid (NanoColl) intracutaneously the day before surgery. Ten minutes before the start of surgery, 0.5–1.0 ml of Patent Blue was injected intracutaneously in the same quadrant of the breast as the tumor was located. The SLN was detected by a gamma probe, and nodes that were hot or blue stained were sent for analysis. If the TIC test was positive, a CLND was performed in the same procedure.

### Pathologic examination

The sentinel lymph node was bisected along the long axis. Two imprints were made from each SLN by touching the cut surface of the SLN to a glass slide. An immunocytochemical staining was used to detect breast cancer metastasis on the imprint with the help of a cytokeratin antibody (MNF-116) on one imprint glass while the other imprint glass was stained using the May-Grünwald-Giemsa staining method. A microscopical interpretation of both imprint glasses was rendered by a board-certified pathologist. The SLN was then fixed in 10% formalin, processed in the usual manner, and paraffin embedded. Four levels of each halves of the SLN were cut from the paraffin block at 50-micrometer intervals, which resulted in eight H&E levels to be examined histopathologically. Intraoperative pathological diagnostic categories on TIC included only either positive (metastasis) or negative (absence of tumor cells) result in the SLN. No “suspicious” statement as an answer was accepted from the pathologist. The surgical team was immediately informed about the pathological result by phone.

### Statistics

Data analysis was performed using SPSS version 24. Chi-square and Fisher exact tests were used to assess the association between pathologic factors and TIC results. Sensitivity was defined as the number of patients who are both disease positive (with positive sentinel lymph nodes on definitive histology) and test positive (positive TIC results), so called true positives (TP), divided by the number who are disease positive (both true positives and false negatives (FN)). Sensitivity = TP / (TP + FN). Specificity was defined as the number who are both disease negative (with negative sentinel lymph nodes on definitive histology) and test negative (negative TIC results), so called true negatives (TN), divided by the number who are disease negative (both true negatives and false positives (FP)). Specificity = TN / (TN + FP). Statistical significance was defined as a p-value of less than 0.05.

## Results

A total of 1227 patients were included in the analysis (Table 1). From this cohort we identified 885 patients (72.1%) with an invasive ductal carcinoma, 161 patients (13.1%) with an invasive lobular carcinoma, 97 patients (7.9%) with an invasive tubular carcinoma and 84 patients (6.9%) with other types of breast carcinoma. The mean age was 60 years (range 24–90 years). The median tumor size was 19.0 mm (range 1.5–150.0 mm) and 725 patients (59.1%) had T1 tumors, 439 patients (35.8%) had T2 tumors and 63 patients (5.1%) had T3 tumors. Breast conserving therapy was performed in 903 patients (73.6%).

**Table 1:** Patient and tumor characteristics.

Characteristic (n = 1227)	n (range or %)
Mean age	60 years (24–90)
Type of surgery	
*Breast conserving surgery*	903 (73.6)
*Mastectomy*	324 (26.4)
Median tumor size	19.0 mm (1.5–150.0)
T stage	
*T1*	725 (59.1)
*T2*	439 (35.8)
*T3*	63 (5.1)
Tumor histology	
*Invasive ductal carcinoma*	885 (72.1)
*Invasive lobular carcinoma*	161 (13.1)
*Invasive tubular carcinoma*	97 (7.9)
*Other*	84 (6.9)
Nuclear grade (n = 1224)	
*Low*	307 (25.1)
*Intermediate*	630 (51.5)
*High*	287 (23.4)
ER Positive (n = 1226)	1076 (87.8)
PR Positive	791 (64.5)
HER-2/neu status (n = 1226)	
Positive	148 (12.1)
Negative	1078 (87.9)

ER estrogen receptor; PR progesterone receptor

From these 1227 patients, a total of 1974 SLNs were evaluated by intraoperative TIC (on average, 1.61 SLNs per patient). 280 patients (22.8%) had metastasis in SLNs of which 6 were missing information on size of the metastasis. 12 patients (4.4%) had isolated tumor cells (ITC), 46 patients (16.8%) had micrometastases (≤2mm) and 216 patients (78.8%) had macrometastases (>2mm).

A total of 280 patients had positive SLNs on definitive histology, of these 88 were falsely negative by TIC (sensitivity 68.6%). Two patients with a positive TIC had a negative SLN on definitive histology (specificity 99.8%). In the first case the MNF-116 antibody was negative for staining but as the pathologist suspected metastatic cells on microscopic examination the decision was made to do a CLND during the operation, definitive examination showed no metastasic cells. In the second case the MNF-116 antibody was negative for staining but as it was positive for giemsa staining the test was interpreted as positive by the pathologist, despite this the operating surgeon interpreted the answer as inconclusive and decided to await definitive answer and an immediate CLND was not performed. On definitive pathological examination there were no signs of metastatic cells. Both of these patients were diagnosed in the first year of using the method.

The sensitivity of TIC according to histological subtype was as following, invasive ductal carcinoma was 69.7% (CI 63.6%-75.8%), for invasive lobular carcinoma 73.7% (CI 59.7%-87.7%) and for invasive tubular carcinoma 52.2% (CI 31.8%-72.6%). The sensitivity by T-stage was T1a = 100%, T1b = 62.5% (CI 47.2%-77.8%), T1c = 54.4% (CI 46.2%-62.6%), T2 = 73.3% (CI 64.3%-82.3%) and T3 = 88.2% (CI 63.8%-100%). The sensitivity increased over the study period, from 56.3% in 2003 to 83.1% in 2008 (Table 2).

**Table 2:** Touch imprint cytology (TIC) sensitivity.

	Overall (%)	Ductal (%)	Lobular (%)	Tubular (%)
**Age**				
*Age < 50 years (n = 258)*	65.2	69.0	62.5	100
*Age ≥ 50 years (n = 967)*	70.1	69.9	76.7	60.0
**T stage**				
*T1a (n = 38)*	100	100	100	100
*T1b (n = 154)*	62.5	75.0	100	100
*T1c (n = 534)*	54.4	54.5	70.0	38.5
*T2 (n = 437)*	73.3	73.6	77.8	57.1
*T3 (n = 62)*	88.2	88.0	87.5	100
**Year of operation**				
*2004 (n = 247)*	56.3	56.4	25.0	80.0
*2005 (n = 183)*	56.8	60.0	63.6	33.3
*2006 (n = 243)*	54.4	54.5	70.0	38.5
*2007 (n = 235)*	73.3	73.6	77.8	57.1
*2008 (n = 317)*	88.2	88.0	87.5	100

The relative number of macrometastases varied according to tumor type, ductal carcinoma 77.1% (CI 71.5%-82.8%), lobular carcinoma 94.7% (CI 87.3%-102%) and tubular carcinoma 69.2% (CI 44.1%-94.3%). Comparing the groups of false negative versus true positive TIC shows similarities between age, tumor type, nuclear grade as well as ER and PR status. The false negative group had smaller tumor size, T1 50.0% vs 28.2%, fewer HER2 positive, 6.8% vs 17.9% and fewer patients with macrometastases 55.7% vs 89.2% (Table 3).

**Table 3:** Characteristics of false negative and true positive touch imprint cytology (TIC).

	False negative (n = 88)	True positive (n = 195)
**Characteristic**	n (%; 95%CI)	n (%; 95%CI)
**Tumor type**		
*Ductal*	67 (76.1; 67.2–85.0)	154 (80.0; 73.3–84.7)
*Lobular*	10 (11.4; 4.7–18.0)	28 (14.4; 9.4–19.3)
*Tubular*	11 (12.5; 5.6–19.4)	12 (6.2; 2.8–9.5)
**T stage**		
*T1*	44 (50.0; 39.6–60.5)	55 (28.2; 21.9–34.5)
*T2*	40 (45.4; 35.1–55.9)	110 (56.4; 49.4–63.4)
*T3*	4 (4.5; 0.2–8.9)	30 (15.4; 10.3–20.4)
**Grade III**	22 (25.0; 16.0–34.0)	59 (30.3; 23.8–36.7)
**ER positive**	81 (92.0; 86.4–97.7)	174 (89.2; 84.9–93.6)
**PR positive**	62 (70.5; 60.9–80.0)	126 (64.6; 57.9–71.3)
**HER2 positive**	6 (6.8; 1.6–12.1)	35 (17.9; 12.6–23.3)
**SLN metastasis size**		
*Isolated tumor cells (ITC)*	5 (5.7; 0.8–10.5)	7 (3.6; 1.0–6.2)
*Micrometastasis*	34 (38.6; 28.5–48.8)	14 (7.2; 3.6–10.8)
*Macrometastasis*	49 (55.7; 45.3–66.1)	174 (89.2; 84.9–93.6)

## Discussion

The aim of the study was to evaluate the sensitivity and specificity of TIC in a large consecutive cohort of patients with breast carcinoma. Having a high false positive rate is unacceptable because that means that patients will undergo an unnecessary operation of the axilla with a high morbidity rate. This study shows a very low false positive rate with only two patients out of 1227 (0.16%) with false positive test results, giving an overall specificity of 99.8%. The reason for false positive results was misinterpretation of the test by the pathologist as MNF-116 antibody was negative in the two cases. Only one needless CLND was performed, and according to the current guidelines, the operation wouldn't have been performed today because formerly even the presence of isolated tumor cells fulfilled the criteria for a CLND [11]. The specificity also improved over time, with all of the patients having a false positive test results included in the first year of using the method.

The sensitivity for TIC has been previously reported to vary widely from 34% to 96% [9]. The sensitivity for ductal and lobular carcinoma was acceptably high at 69.7% and 73.7% respectively, it was considerably lower for tubular cancer (52.2%) but the differences between the tumor types was not statistically significant. Despite this, it has not been shown that the detection of lobular cancer on TIC is more difficult compared to ductal cancer [7,12].

Many of the previous studies included only tumors less than 5 cm in size [9,13]. In this study we included T1-T3 tumors and the sensitivity increased in relation to tumor size. For tumors 11–20 mm the sensitivity was 54.4%, for tumors over 50 mm the sensitivity was 88.2%. Teal et al. has shown that the sensitivity of intraoperative evaluation increases as the primary tumor is larger, i.e. the larger the tumor is, the higher the risk of a metastatic node involvement becomes [14]. In the T1a group there were no false negative TIC results, presumably because of the small tumor size and low risk for spread to the SLN, which explains the high sensitivity of 100%.

There was also an increase in sensitivity of the method over time, which might be explained by the learning curve of the method. By comparing the false negative and true positive TIC results we did find that the patients who had false negative test results had fewer macrometastasis. The downside of the method is that the SLN is split down the middle, the metastasis would have to be located in that area of the node to be detected, higher detection rate of macrometastasis is expected for that reason.

Because of the surprisingly low rate of axillary recurrence among patients who have not been subjected to any axillary procedure, as well as SLN-positive patients who have not undergone CLND, the role of CLND has increasingly been discussed. Two studies have been published in recent years in which sentinel node positive breast cancer patients were randomized either to undergo CLND or not. The first study (ACOSOG Z0011) included patients with SLN macrometastases who had breast-conserving surgery [15]. The second study (IBCSG 23–01) included patients with SLN micrometastasis [16]. The studies did not demonstrate any difference in the rate of axillary recurrence, and survival was even slightly better among patients who underwent SLN biopsy alone, although the difference was not statistically significant. These findings raise the question of the future role for TIC, since the need for a rapid answer in the operating theater diminishes. However, for patients not fulfilling the inclusion criteria in the Z0011 trial, or for patients where a second surgery entails a high risk for the patient (e.g. old patients with severe co-morbidities or pregnant patients), TIC is still a valuable option with a high specificity and an acceptable sensitivity. The utility of the test is higher in patients with larger sized primary tumors and the test is independent of tumor subtype.

## Supporting information

S1 Dataset TIC_SPSS_dataset. (SAV)
